# Analysis of Interactions of *Salmonella* Type Three Secretion Mutants with 3-D Intestinal Epithelial Cells

**DOI:** 10.1371/journal.pone.0015750

**Published:** 2010-12-29

**Authors:** Andrea L. Radtke, James W. Wilson, Shameema Sarker, Cheryl A. Nickerson

**Affiliations:** 1 School of Life Sciences, Center for Infectious Diseases and Vaccinology, The Biodesign Institute at Arizona State University, Tempe, Arizona, United States of America; 2 Department of Biology, Villanova University, Villanova, Pennsylvania, United States of America; Charité-University Medicine Berlin, Germany

## Abstract

The prevailing paradigm of *Salmonella* enteropathogenesis based on monolayers asserts that *Salmonella* pathogenicity island-1 Type Three Secretion System (SPI-1 T3SS) is required for bacterial invasion into intestinal epithelium. However, little is known about the role of SPI-1 in mediating gastrointestinal disease in humans. Recently, SPI-1 deficient nontyphoidal *Salmonella* strains were isolated from infected humans and animals, indicating that SPI-1 is not required to cause enteropathogenesis and demonstrating the need for more *in vivo-*like models. Here, we utilized a previously characterized 3-D organotypic model of human intestinal epithelium to elucidate the role of all characterized *Salmonella enterica* T3SSs. Similar to *in vivo* reports, the *Salmonella* SPI-1 T3SS was not required to invade 3-D intestinal cells. Additionally, *Salmonella* strains carrying single (SPI-1 or SPI-2), double (SPI-1/2) and complete T3SS knockout (SPI-1/SPI-2: *flhDC*) also invaded 3-D intestinal cells to wildtype levels. Invasion of wildtype and TTSS mutants was a *Salmonella* active process, whereas non-invasive bacterial strains, bacterial size beads, and heat-killed *Salmonella* did not invade 3-D cells. Wildtype and T3SS mutants did not preferentially target different cell types identified within the 3-D intestinal aggregates, including M-cells/M-like cells, enterocytes, or Paneth cells. Moreover, each T3SS was necessary for substantial intracellular bacterial replication within 3-D cells. Collectively, these results indicate that T3SSs are dispensable for *Salmonella* invasion into highly differentiated 3-D models of human intestinal epithelial cells, but are required for intracellular bacterial growth, paralleling *in vivo* infection observations and demonstrating the utility of these models in predicting *in vivo*-like pathogenic mechanisms.

## Introduction

Infections with nontyphoidal *Salmonella* serovars are a leading cause of bacterial enteric disease and are the single most common cause of death from food-borne illnesses in the United States [1]. An essential feature of the pathogenicity of *Salmonella* is its interaction with host intestinal epithelial cells, where *Salmonella* invasion/entry into host cells is critical for bacterial survival and establishment of disease in a host. Currently, it is thought that *Salmonella* invasion of epithelial cells requires the Type Three Secretion System (T3SS), a Gram negative bacterial “molecular syringe” that injects effector proteins into the host cell cytosol, thus altering cellular functions [2], [3], [4]. *Salmonella* possesses two T3SSs that direct the secretion of proteins into host cells, and a third T3SS that directs the secretion of proteins to be assembled into the external flagellular organelle [5], [6]. The T3SS considered to be necessary for epithelial cell invasion is encoded on Salmonella Pathogenicity Island-1 (SPI-1), a large region of the *Salmonella* genome that contains numerous genes involved in pathogenesis and infection [6], [7], [8], [9]. The second T3SS is encoded on a second pathogenicity island, SPI-2, which is induced after *Salmonella* invasion of host cells and secretes protein effectors necessary for intracellular bacterial trafficking and replication [10], [11]. The third T3SS is under the transcriptional control of the *flhDC* operon, the master regulator of the flagellum assembly machinery [5], [12], [13], [14].

A significant portion of our knowledge regarding *Salmonella enterica* serovar Typhimurium (hereafter referred to as *Salmonella)* T3SSs and their role in infection of epithelial cells has been derived from studies that utilized non-polarized monolayer cell culture systems [6], [10], [15]. However, during *in vivo* infections, *Salmonella* infects polarized epithelial cells of the host intestinal tract [16]. In recognition that conventional monolayer cell culture models do not display the structure, morphology, and architecture of the intestinal tract, more advanced and *in vivo-*like model systems have been developed and implemented to study *Salmonella* pathogenesis, which include polarized intestinal cell culture models, ileal loop models, *C. elegans* model, and mouse, cattle, and chick animal models [17], [18], [19], [20], [21], [22], [23], [24], [25], [26]. Studies utilizing these more complex model systems demonstrated that SPI-1 was critical in the establishment of a *Salmonella* infection. While these model systems have certainly unveiled critical insight into the lifecycle and pathogenic mechanisms of *Salmonella*, the majority of these studies have utilized non-human model systems to predict what potentially could be occurring during infection of humans. Moreover, it was shown that the severity and role of SPI-1 and 2 to cause infection by *Salmonella* Typhimurium depends on the host species, which further complicates data extrapolation to the human condition [21], [23], [27]. The importance of host species in investigating and understanding the mechanisms of *Salmonella*-induced gastrointestinal disease was further demonstrated in a recent key study looking at a naturally occurring *Salmonella* outbreak in humans, which revealed conflicting results as compared to monolayers and non-human animal models [28]. Specifically, the study identified two clinical isolates of *Salmonella enterica* serovar Senftenberg present in stool samples from a food-borne human disease outbreak that lacked essential SPI-1 T3SS structural components and SPI-1 encoded secreted effectors [28]. Infection of mice with these isolated strains displayed little to no intestinal pathology or inflammation, suggesting these bacterial strains are limited to cause only human enteropathology. Furthermore, these naturally occurring SPI-1 deficient strains invaded human monolayer cells less efficiently than laboratory constructed SPI-1 mutants, further demonstrating differences between monolayer systems and *in vivo* outcomes of infection.

**Figure 1:**
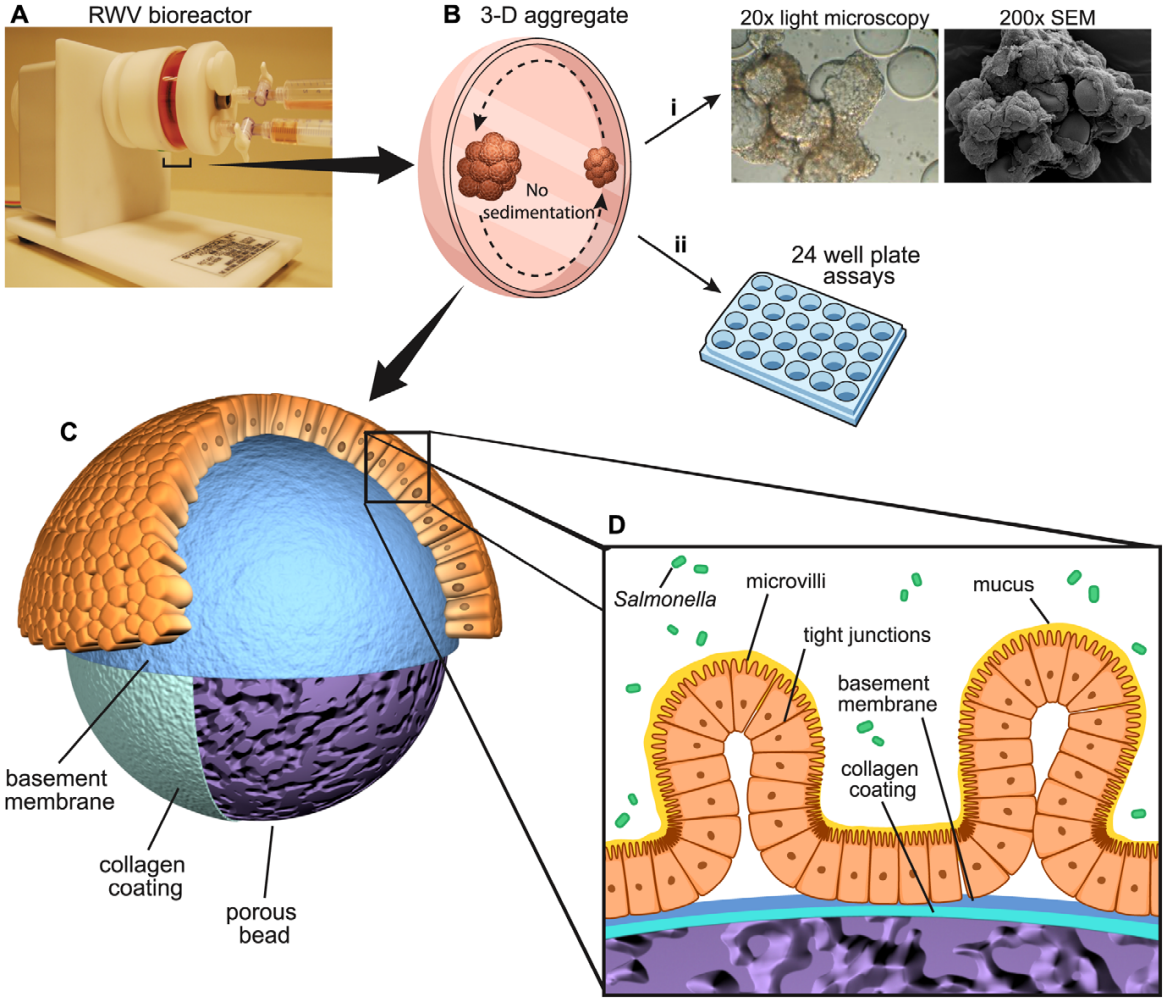
Depiction and operation of the RWV cell culture system. A) Rotating Wall Vessel (RWV) bioreactor containing cell culture media and porous microcarrier beads. B) Sedimentation of cells within the RWV is offset by the rotating fluid, creating a constant free fall of the cells through the culture medium, allowing the cells to form 3-D aggregates under conditions of physiological fluid shear. Three-dimensional aggregates are taken out of the bioreactor for analysis including light microscopy and Scanning Electron Microscopy (SEM) (i), or seeded evenly in a 24 well plate format for various profiling and infection assays (ii). C) Enlarged representation of intestinal epithelial cells attached to microcarrier beads of a 3-D aggregate. After intestinal epithelial cells adhere to the beads, they begin to differentiate and display *in vivo*-like characteristics such as narrow columnar cellular morphology, basement membrane, tight junctions, mucus, and microvilli [31], [32], [36]. D) A further enlarged depiction of a single bead within the 3-D aggregate displaying characterized cellular surface structures and components, and infected with *Salmonella*.

Collectively, these studies demonstrate how different *Salmonella* infection model systems can give conflicting results, with the most dependable and accurate model being the natural host. Unfortunately, a human *Salmonella* infection model is unrealistic and impractical, in turn the next best model system would be one that best approximates the parental tissue *in vivo*. To achieve an *in vitro* cell culture model that more closely mimics *in vivo* cellular characteristics, including three-dimensional architecture, multicellular complexity, mucus production, apical/basolateral polarity, and well developed tight junction formation [29], our laboratory utilizes the Rotating-Wall Vessel (RWV) bioreactor system (Figure 1). The gentle fluid rotation of the RWV creates a physiological low fluid shear environment that allows the cells to grow in three-dimensions, aggregate based on natural cellular affinities, and differentiate into human surrogate tissue-like models possessing *in vivo* characteristics not observed in the same cells grown as monolayers [30], [31], [32], [33], [34], [35], [36]. In this study we use a RWV-generated organotypic 3-D model derived from the colon epithelial cell line, HT-29. HT-29 cells were chosen as the model cell line in this study because a) they have been extensively characterized in 3-D and shown to be a physiologically relevant model of the parental tissue from which they were derived [32], b) they represent the primary site of *Salmonella* intestinal infection [37], c) we have previously applied this system to model fundamental aspects of the host-pathogen interactions with *Salmonella* [32], and d) numerous studies have been conducted using this cell line grown as monolayers to study the initial stages of the host-pathogen interaction between *Salmonella* and intestinal epithelium that lead to gastrointestinal disease [18], [32], [38].

In this work, we demonstrate several key aspects of *Salmonella* pathogenesis during infection of RWV-derived 3-D organotypic models of human colonic epithelium that mimic *in vivo* observations, but contradict prevailing paradigms established using monolayers and many non-human model systems. We report for the first time that *Salmonella* is able to invade 3-D intestinal cells independently of all known *Salmonella* T3SSs, including SPI-1 and SPI-2 encoded T3SSs and the flagellar secretory system regulated by the *flhDC* operon [13]. Within the 3-D intestinal epithelial aggregates, we were able to identify three different epithelial cell types that are present in the human large intestine, further showing the complexity and physiological relevance of the 3-D intestinal model. Unlike previous *in vitro* reports, there was no identifiable preferential host cell type targeted in our 3-D intestinal models during infection by wildtype or the T3SS *Salmonella* mutants [39], [40], [41], [42]. Non-invasive bacterial strains, bacterial-size beads, and heat-killed *Salmonella* did not appear to be localized intracellularly in 3-D intestinal cells, demonstrating that an active bacterial invasion mechanism(s) is necessary for internalization, rather than a non-specific host cellular uptake mechanism. Following invasion of 3-D cells, the SPI-1, SPI-2, SPI-1/2 double mutant, and the SPI-1/2 *flhDC* triple T3SS mutant, all grew to similar levels intracellularly, but to substantially lower levels than wildtype *Salmonella*. Collectively, we provide evidence that our highly differentiated 3-D models of human colonic epithelium exhibit infection phenotypes that are consistent with an *in vivo* enteric *Salmonella* infection. To our knowledge, this is the first report that bacterial invasion, but not intracellular growth, is largely independent of the functions encoded by TTSSs.

## Results

### *Salmonella* invades 3-D intestinal cells in the absence of SPI-1, SPI-2, and flagellum T3SSs and their effector proteins

**Figure 2:**
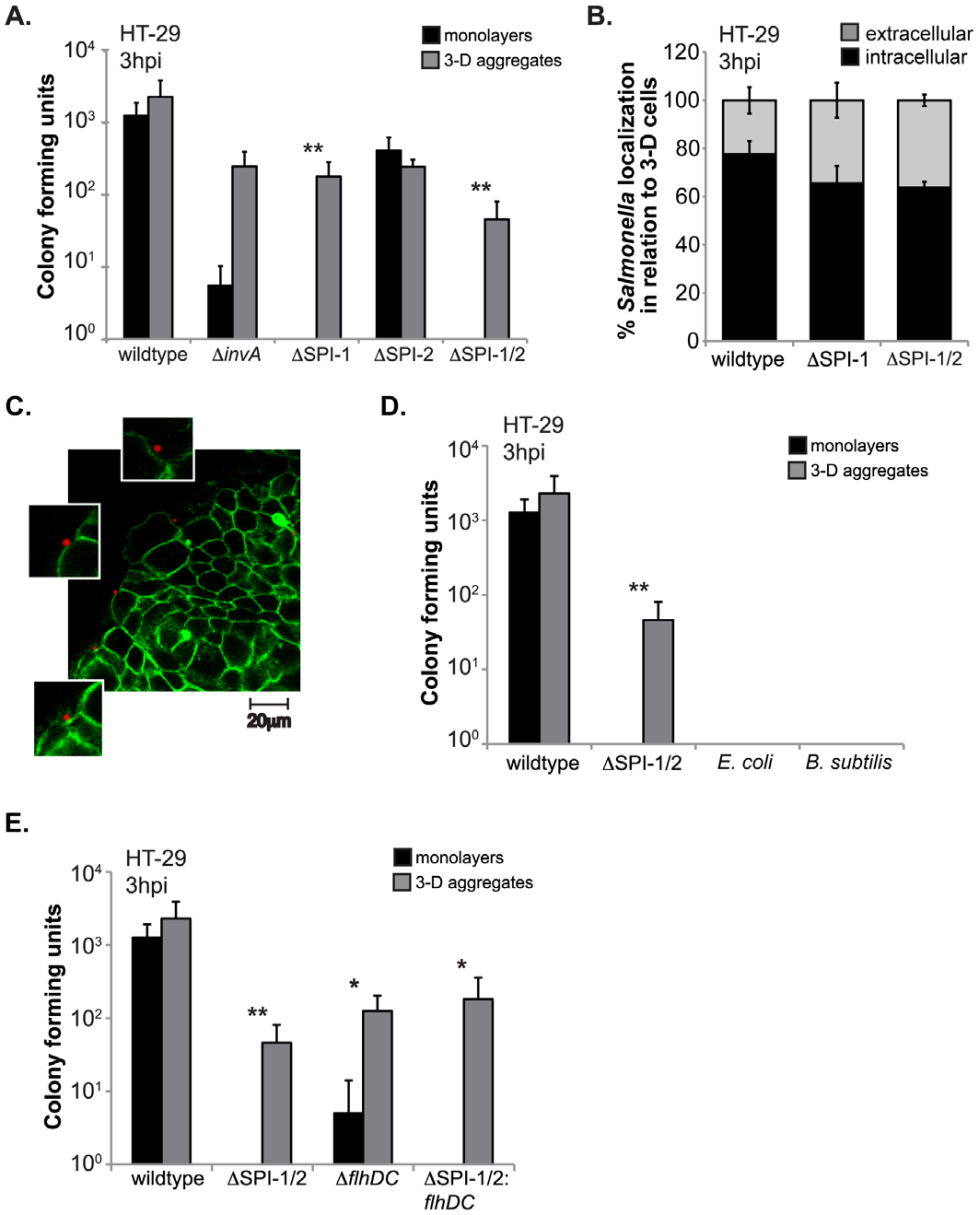
SPI-1, SPI-2, and the flagellar secretory system are not required for *Salmonella* invasion into 3-D intestinal cells. A) HT-29 monolayers (black bars) and 3-D aggregates (grey bars) infected with wildtype, Δ*invA*, ΔSPI-1, ΔSPI-2, and ΔSPI-1/2 *Salmonella* mutants for 1 hour at an m.o.i. of 10. To measure bacterial invasion, host cells were lysed at 3 hpi and intracellular bacteria were enumerated and plotted as CFU obtained for each bacterium. B) Three-dimensional HT-29 cells were infected as in (A), fixed at 3 hpi, stained with a *Salmonella* monoclonal antibody (extracellular bacteria), washed, permeabilized, stained with a *Salmonella* polyclonal antibody (extracellular and intracellular bacteria), and imaged by immunofluorescence confocal microscopy. Percentages represent number of bacteria out of ∼50 bacteria that stained with the monoclonal antibody pre-permeabilization (grey bars) or the remaining bacteria that stained with the polyclonal antibody and not the monoclonal antibody post-permeabilization (black bars). C) Representative single image from z-stack confocal immunofluorescence microscopy images (100× objective) of 3-D HT-29 aggregates exposed to red fluorescent beads (2 µm) for 1 hour, washed, fixed 3 hours post exposure, and stained with phalloidin antibody to visualize host cell actin (green). D) HT-29 monolayers (black bars) and 3-D aggregates (grey bars) infected with wildtype *Salmonella*, ΔSPI-1/2 *Salmonella*, *E. coli,* and *B. subtilis* for 1 hour at an m.o.i. of 10. To measure bacterial invasion, host cells were lysed at 3 hpi and intracellular bacteria were enumerated and plotted as CFU obtained for each bacterium. E) HT-29 monolayers (black bars) and 3-D aggregates (grey bars) infected with wildtype, ΔSPI-1/2, Δ*flhDC*, ΔSPI-1/2: *flhDC Salmonella* mutants for 1 hour at an m.o.i. of 10. To measure bacterial invasion, host cells were lysed at 3 hpi and intracellular bacteria were enumerated and plotted as CFU obtained for each bacterium. Data represents the average of at least three independent experiments from separate batches of cells (N = 3). Limit of detection is 10 CFU. * and ** indicate statistical significance of *p*<0.05 and 0.001, respectively, comparing 3-D aggregates to monolayers for each bacterial strain.

In previous reports from our lab utilizing classic gentamicin invasion assays, we showed that a *Salmonella invA* mutant that is defective for invading monolayers, exhibited wildtype levels of invasion into 3-D HT-29 colon cells [32], [43]. *InvA* is encoded by SPI-1 and is essential in the formation of the T3SS needle. However, SPI-1 encodes for numerous other genes aside from *invA* that are involved in bacterial invasion and virulence [2]. To investigate if alternative SPI-1 encoded effector protein(s) are potentially responsible for *Salmonella* invasion into 3-D intestinal cells, we infected HT-29 monolayers and 3-D aggregates with either a *Salmonella* mutant lacking the *invA* gene or a mutant lacking the entire SPI-1 encoding region (Figure 2A). The *invA* mutant and the SPI-1 mutant both invaded 3-D HT-29 cells to higher levels compared to monolayers. These data suggest that SPI-1, a virulence factor previously identified to be required for *Salmonella* invasion of epithelial cells grown as monolayers [9], [44], is not mandatory for invasion into our well-differentiated 3-D intestinal epithelial cells.

We next wanted to test the hypothesis that the second T3SS encoded by SPI-2 and/or its encoded effectors were potentially compensating for the loss of SPI-1, thereby aiding in the invasion of *Salmonella* into the 3-D cells. To address this question, we infected HT-29 monolayers and 3-D cells with a SPI-2 deletion mutant and a SPI-1/SPI-2 double mutant (Figure 2A). The double mutant was still able to invade 3-D cells to significantly higher levels compared to monolayers, and to similar levels observed for SPI-1 and SPI-2 mutants in 3-D cells. Overall, these data demonstrate that unlike monolayers, *Salmonella* can invade the more *in vivo-*like 3-D cells in the absence of SPI-1 and SPI-2 T3SSs and their encoded effectors. However, the SPI-1 and SPI-2 genomic regions do contribute to a more productive invasion, as wildtype *Salmonella* exhibited significantly higher levels of invasion compared to the T3SS mutants in 3-D cells.

Three-dimensional HT-29 cells were previously shown to secrete mucus, which could cause bacteria to adhere to the surface of the cells and not actually be localized intracellularly [32]. Thus, to further validate that wildtype and the SPI-1 mutant are indeed localized inside 3-D HT-29 cells, we performed a differential immunofluorescence microscopy staining technique that allows one to distinguish between extra- and intracellular bacteria [45], [46]. Three-dimensional HT-29 cells were infected with wildtype and *Salmonella* SPI-1 and SPI-1/2 mutants as in (A), fixed, and stained with a monoclonal anti-*Salmonella* antibody that will bind only extracellular bacteria. After addition of corresponding secondary antibody, cells were washed, permeabilized, and stained with a polyclonal *Salmonella* antibody that can bind to intracellular and extracellular bacteria as a result of host cell membrane permeabilization (Figure S1). Three-dimensional cells were visualized by confocal immunofluorescence microscopy and the bacteria that only stained with the monoclonal antibody (pre-permeabilization/extracellular bacteria) were counted out of 50 total bacteria (stained with both antibodies) in at least three independent experiments (Figure 2B). A majority of the wildtype and *Salmonella* T3SS mutants were localized intracellularly (>60% of total bacteria counted), and no significant difference in bacterial localization was observed between wildtype and T3SS mutants, further demonstrating that *Salmonella* can invade into 3-D HT-29 cells in the absence of SPI-1 and SPI-2 T3SSs.

As shown, *Salmonella* SPI-1 and 2 T3SSs mutants can invade 3-D HT-29 cells; however, we wanted to investigate if *Salmonella* was entering 3-D HT-29 cells through non-specific cellular uptake and not a *Salmonella* active invasion mechanism. To test if 3-D cells are capable of particle uptake, we introduced bacterial sized fluorescent beads to the cells under the identical experimental conditions used for our *Salmonella* infection assays. Three hours post bead exposure; cells were fixed, stained with phalloidin to visualize host cell actin cytoskeleton, and imaged by confocal immunofluorescence microscopy (Figure 2C). The only beads observed were those that appeared to be extracellular in localization. The extracellular localization was further confirmed by z-stack confocal immunofluorescence microscopy (Figure S2).

Next we wanted to investigate if bacterial surface component(s) and/or bacterial active process(s) are required to be present for cellular uptake. We thus infected 3D HT-29 cells with non-invasive Gram negative *E. coli* and Gram positive *B. subtilis* strains, which represent bacteria that have very different surface structures and do not posses mechanisms necessary for host cell invasion. Three hours post infection, we observed no intracellular bacteria by gentamicin survival assay or confocal microscopy (Figure 2D and data not shown). These data suggest that either an invasion mechanism is necessary for internalization, or alternatively, a *Salmonella* specific surface component/structure is triggering *Salmonella* internalization by 3-D HT-29 cells. To determine between these two possibilities, monolayer and 3-D HT-29 cells were infected with heat-killed wildtype *Salmonella* for 1 hour, washed, and fixed. Cells were stained with anti-*Salmonella* antibody, phalloidin, and DAPI to visualize host and bacterial nuclei, and visualized by confocal microscopy using z-stack imaging. We observed no intracellular *Salmonella* in either monolayer or 3-D HT-29 cells (data not shown). Collectively, these data suggest that *Salmonella* is actively invading 3-D HT-29 cells utilizing a mechanism(s) other than the SPI-1 or 2 encoded T3SSs.

Another possible means of *Salmonella* protein effector secretion that could potentiate invasion and allow *Salmonella* active entry into 3-D cells is through a pore created in the bacterial membrane as a result of flagellum translocation to the surface, which occurs through a third T3SS [47], [48]. To test the hypothesis that secreted effector(s) responsible for invasion of the *Salmonella* SPI-1/2 double mutant into 3-D cells is a result of the flagellum pore; we infected cells with a *Salmonella* mutant deficient in SPI-1, SPI-2, and the master regulators of flagellum pore formation and synthesis, FlhC and FlhD (Figure 2E). The SPI-1/SPI-2: *flhDC* mutant was still able to significantly invade the 3-D HT-29 aggregates, demonstrating that *Salmonella* can actively invade 3-D intestinal cells in the absence of all known/characterized T3SSs.

### *Salmonella* does not preferentially target M-cells/M-like cells in 3-D HT-29 models

**Figure 3:**
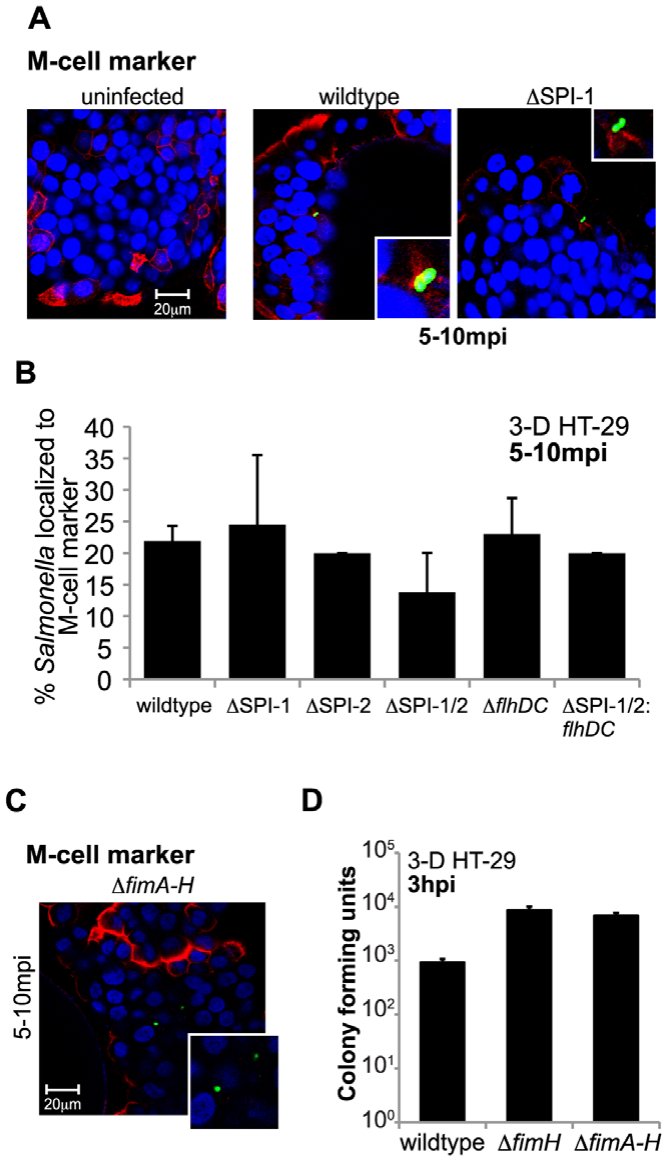
Internalization of wildtype or T3SS mutants into 3-D intestinal cells is not exclusively dependent on M-cells/M-like cells. A) Confocal immunofluorescence microscopy images (100× objective) of 3-D HT-29 aggregates left uninfected or infected for 5–10 min with either the wildtype or SPI-1 *Salmonella* mutant and stained with anti-sialyl Lewis A (M-cell marker) antibody (red), anti-*Salmonella* antibody (green), and DAPI (blue). B) Quantitation of confocal images as observed in (A) of 3-D HT-29 aggregates infected for 5–10 min with wildtype and the T3SS mutants. Percentages represent the number of bacteria localized to M-cell marker/∼50 bacteria counted (N≥3). No significant difference was observed when comparing each bacterial strain to wildtype for each host cell marker. C) Confocal immunofluorescence microscopy images (100× objective) of 3-D HT-29 aggregates infected with a *Salmonella* type 1 pili mutant (Δ*fimA-H*) and stained with anti-sialyl Lewis A antibody (red), anti-*Salmonella* antibody (green), and DAPI (blue). D) HT-29 3-D aggregates infected with wildtype, Δ*fimH*, and Δ*fimA-H Salmonella* mutants for 1 hour at an m.o.i. of 10. To measure bacterial invasion, host cells were lysed at 3 hpi and intracellular bacteria were enumerated and plotted as CFU obtained for each bacterium (N≥3).

The work presented here is the first to report that all known T3SSs are not mandatory for *Salmonella* invasion into epithelial cells, which is consistent with reports of SPI-1 deficient strains being isolated from humans with enteric salmonellosis and animal intestinal tissues [28], [49]. Moreover, a *Salmonella* SPI-1 mutant has also been shown to preferentially target and invade mouse epithelial M cells and polarized epithelial cells cultured under conditions that allow them to express M-cell like phenotypes, phenotypes that include particle uptake and transportation of antigens [16], [39], [41], [50], [51], [52], [53], [54]. From these studies, we hypothesized that *Salmonella* and the T3SS mutants are targeting M-cells to eventually gain entry into the 3-D aggregates. To first observe if the aggregates contained M-cells or M-cell like cells, we stained uninfected 3-D HT-29 cells with anti-sialyl Lewis A antigen antibody (M-cell marker) and imaged the cells by confocal immunofluorescence microscopy (Figure 3A). Interestingly, the 3-D aggregates exhibited very similar staining and expression patterns of the M-cell marker as observed in normal human colon tissue sections [55]. Specifically, the 3-D cells displayed a columnar morphology to where only the edges of >20% of the cells showed expression of the M-cell marker. We next wanted to observe if wildtype and the SPI-1 mutant preferentially targeted M-cells/M-like cells present in the 3-D aggregates to eventually gain intracellular localization (Figure 3A). Three-dimensional HT-29 cells were infected with either wildtype or the *Salmonella* SPI-1 mutant for ∼5 minutes at an m.o.i. of 10∶1 according to the infection protocol outlined by Steele-Mortimer *et al*., 1999 [56]. This established protocol allows for a shorter infection time, a more synchronized infection, and earlier cellular fixation periods, while maintaining the same *Salmonella* intracellular growth and trafficking profiles observed with longer and more commonly used infection protocols (Figure S3A-C) [56]. Wildtype *Salmonella* SPI-1 induction during the different growth conditions utilized in these studies was also analyzed, and no observable or significant differences in SPI-1 expression was seen by RT-PCR and quantitative RT-PCR, respectively (Figure S3D, Text S1, and data not shown). After infection, cells were fixed 5–10 mpi to capture the early stages of infection, and stained with anti-sialyl Lewis A antigen antibody (M-cell marker), anti-*Salmonella* antibody, and DAPI to visualize nuclei (Figures 3A, S4, and S5) [55]. Quantitation by confocal microscopy of the number of wildtype and SPI-1 mutant bacteria out of 50 bacteria in at least 3 independent experiments that localized to cells expressing M-cell marker revealed only 20–25% of wildtype and SPI-1 mutant localized to M-cells/M-like cells, and similar levels were also observed with the wildtype, SPI-2, SPI-1/2, and SPI-1/2: *flhDC* strains (Figure 3B). These data suggest that there is no preferential targeting of wildtype *Salmonella* and the T3SS mutants to M-cells/M-like cells within the 3-D aggregates.

As shown in Figure 3B, *Salmonella* T3SSs are not needed for localization to 3-D HT-29 M-cells/M-like cells, however recent reports suggest that FimH, a component of type I pili, interacts with an M-cell surface protein and this interaction is necessary for *Salmonella* intestinal invasion specifically into M-cells [42], [57]. To determine if the type I pili is playing a role in *Salmonella* targeting and internalization into 3-D aggregates, and more specifically M-cells, we infected 3-D HT-29 cells with a type I pili mutant (ΔFimA-H). Consistent with reports that type I pili is needed to bind to M-cells in order to invade these cell types; we observed no co-localization of *Salmonella* pili mutant with the M-cell marker at 5-10 mpi by confocal immunofluorescence (Figure 3C). However, both the mutant lacking entire pili (Δ*fimA-H*) and the mutant lacking just the adhesion portion of the pili (Δ*fimH*), were still able to invade 3-D cells to similar levels as wildtype (Figure 3D). Utilization of the Fim mutants is also functioning as a control for bacteria that cannot interact with M-cells, but can still invade our 3-D aggregates, demonstrating that *Salmonella* entry into 3-D cells is not solely dependent upon interactions with M-cell/M-like cells.

### *Salmonella* T3SS mutants target multiple intestinal epithelial cell types within 3-D aggregates

**Figure 4:**
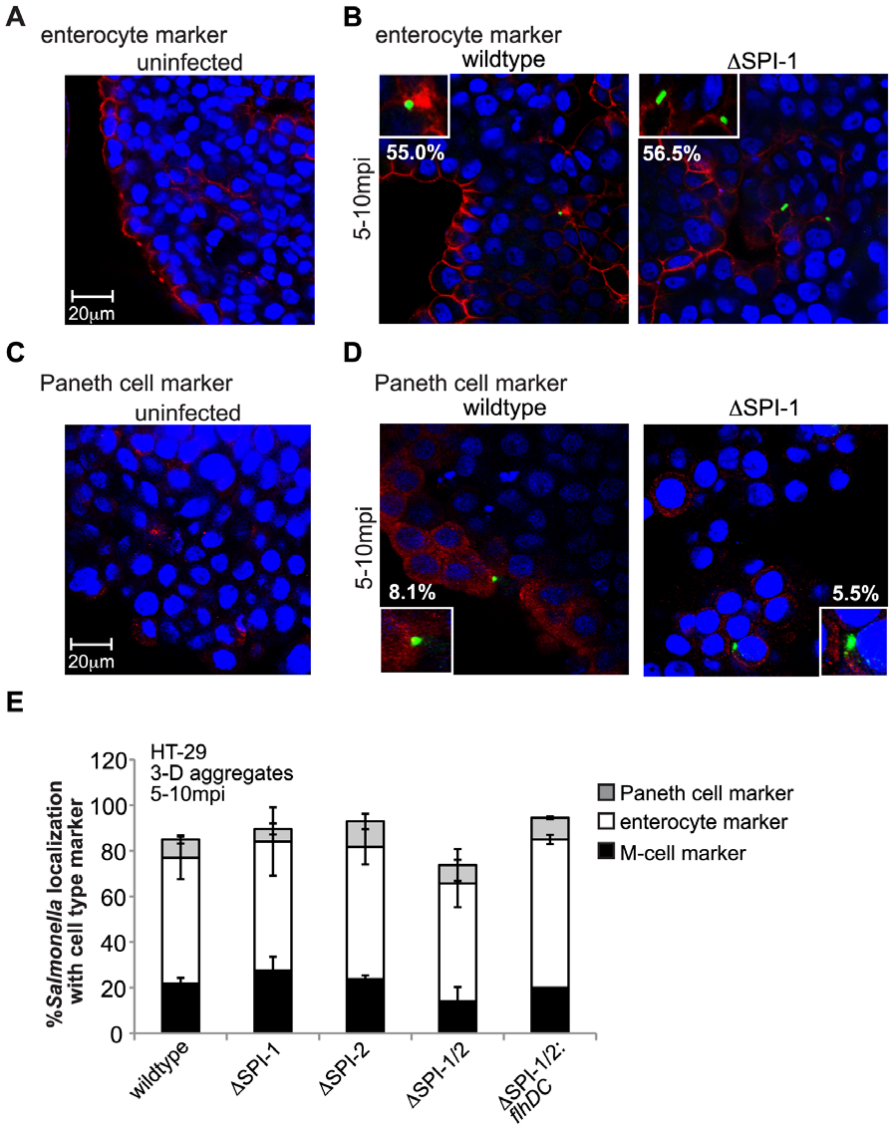
No preferential host cell type is targeted by wildtype and T3SS *Salmonella* mutants during infection of 3-D intestinal cells. A) Uninfected 3-D HT-29 cells fixed and stained with anti-syndecan-1 antibody (enterocyte marker) and DAPI to visualize nuclei. B) Confocal immunofluorescence microscopy images (100× objective) of 3-D HT-29 aggregates infected with either wildtype or SPI-1 *Salmonella* mutant, fixed 5 mpi, and stained with enterocyte marker (red), anti-*Salmonella* antibody (green), and DAPI (blue). Percentages represent bacteria localized to enterocyte marker (N = 3). C) Uninfected 3-D HT-29 cells fixed and stained with anti-PLA2 antibody (Paneth cell marker) and DAPI. D) Confocal immunofluorescence microscopy images (100× objective) of 3-D HT-29 aggregates infected with either wildtype or SPI-1 *Salmonella* mutant, fixed 5 mpi, and stained with Paneth cell marker antibody (red), anti-*Salmonella* antibody (green), and DAPI (blue). Percentages represent number of bacteria localized to Paneth cell marker (N = 3). E) Quantitation of confocal images as observed in Figs. 3A, 4B and 4D, and also including 3-D HT-29 aggregates infected for 5-10 min with SPI-2, SPI-1/2, and SPI-1/2: *flhDC Salmonella* mutants and profiled with host cellular markers. Percentages represent the number of bacteria localized to markers/∼50 bacteria from three different experiments and independent batches of 3-D HT-29 cells. No significant difference was observed when comparing each bacterial strain to wildtype for each host cell marker (N = 3).

The large intestine, from which HT-29 cells are derived, is composed of multiple epithelial cell types, and we used confocal microscopy to assess if any of these cell types are present in our 3-D aggregates and if *Salmonella* is targeting these cell types during infection [58]. We identified two other cell types in addition to M-cells/M-like cells using immunofluorescence labeling, enterocytes and Paneth cells (Figures 4 A and C). Since enterocytes make up the majority of cells within the intestine and also have known absorptive properties, we predicted that a significant proportion of *Salmonella* not localized to M-cells/M-like cells could be targeting enterocytes [59]. After dual staining of 3-D HT-29 cells with the M-cell marker (sialyl Lewis A) and enterocyte marker (syndecan-1) antibodies, we observed very little overlap of cells expressing both markers, demonstrating two different cellular populations within the aggregates (Figure S6) [54], [55], [60], [61]. Three-dimensional cells were infected with either wildtype or SPI-1 *Salmonella* mutant, fixed 5 mpi, stained with both enterocyte and *Salmonella* specific antibodies, and the number of bacteria out of ∼50 bacteria localized to the enterocyte marker was quantitated by confocal immunofluorescence (Figure 4B). A majority of bacteria localized to cells expressing the enterocyte marker, with no significant difference in localization between wildtype and the SPI-1 mutant, suggesting that the SPI-1 encoded T3SS does not play a role in host cell type targeting in the intestinal epithelium during infection.

The third cell type we identified in the 3-D HT-29 aggregates were Paneth cells, a cell type known to secrete antimicrobials, including defensins, with no detectable absorptive properties [62], [63], [64], [65] (Figure 4C). After 3-D HT-29 infection with the wildtype or SPI-1 mutant, and quantitation by confocal microscopy of the number of bacteria out of ∼50 bacteria that localized to cells expressing the Paneth cell marker (Phospholipase A2) [66], we observed <10% of bacteria localized to cells expressing this marker (Figure 4D). Consistent with previous reports of diseased colon tissue samples, we noted an increase in Paneth cell marker expression upon infection (Figure 4C and D) [64], [65]. Goblet cells are a known fourth cell type within the intestine. While our 3-D HT-29 cells did exhibit mucus production (a hallmark of goblet cells), we were unable to definitively identify goblet cells in the 3-D HT-29 cells by immunofluorescence microscopy using a goblet cell marker antibody [32], [67]. The *Salmonella* SPI-2, SPI-1/2, and SPI-1/2: *flhDC* mutants were also profiled for host cell type localization within the 3-D HT-29 aggregates, and similar localization profiles with no significant difference in marker localization of these mutants were observed as compared to wildtype *Salmonella* (Figure 4E). Collectively, these data suggest that *Salmonella* T3SSs do not influence the active targeting of the bacteria to a specific cell type in the 3-D intestinal cells during infection.

### *Salmonella* T3SSs are required for substantial intracellular growth in 3-D HT-29 cells

**Figure 5:**
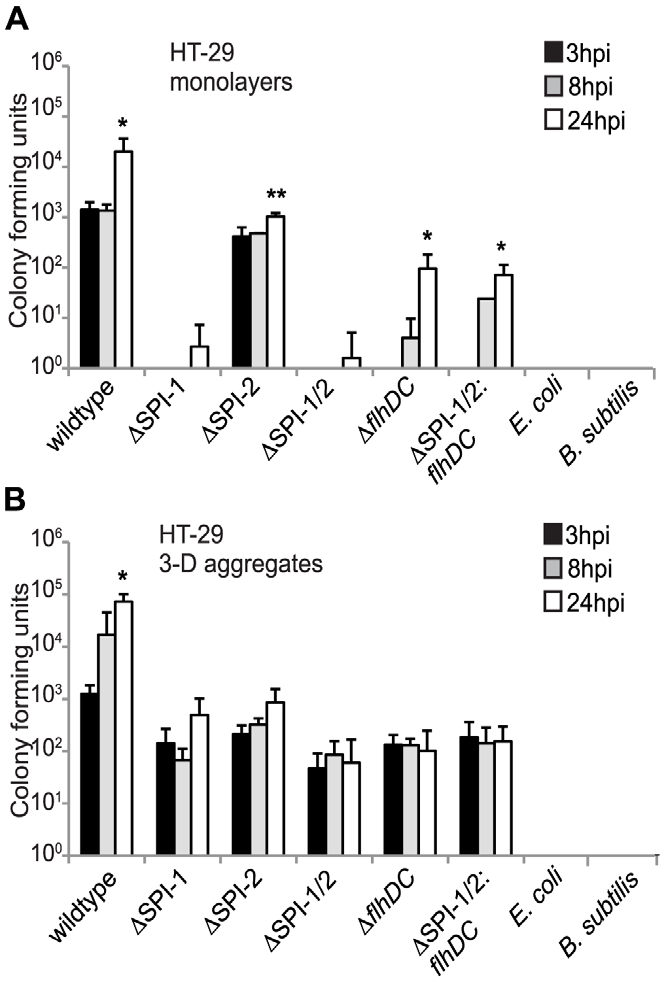
Intracellular growth profiles of *Salmonella* T3SS mutants in HT-29 monolayers and 3-D cells. Intracellular growth of wildtype *Salmonella,* T3SS *Salmonella* mutants, and non-invasive *E. coli* and *B. subtilis* strains measured at 3 hpi (black bars), 8 hpi (gray bars), and 24 hpi (white bars) in HT-29 monolayers (A) and 3-D aggregates (B). For both cell culture models, HT-29 cells were infected at an m.o.i. of 10 for 1 hour and lysed at each indicated time point and plated to enumerate bacterial CFU. CFU quantitation represents an average of at least three independent experiments from three separate batches of cells. * and ** indicate statistical significance of *p*<0.05 and 0.001, respectively, comparing 8 and 24 hpi to 3 hpi for each bacterial strain.

Our data established that *Salmonella* T3SSs are not required for invasion into 3-D HT-29 cells; however we were interested in observing the intracellular growth profiles of the T3SS mutants after entry into 3-D cells and their profiles compared to monolayers (Figure 5). We thus infected monolayers and 3-D HT-29 cells with the various bacterial strains at an m.o.i. of 10 for 1 hour. At the indicated times post infection, host cells were lysed and bacteria were enumerated for colony forming units. Wildtype *Salmonella* and the SPI-2 mutant had similar growth profiles between monolayers and 3-D cells with an increase in growth by 24 hpi compared to 3 hpi. The SPI-1, SPI-1/2, *flhDC*, and SPI-1/2: *flhDC* mutants had little to no detectable growth by 24 hpi in monolayers; however, these mutants grew to levels similar as the SPI-2 mutant in 3-D cells, although the mutant strains never reached wildtype growth levels. The non-invasive Gram negative *E. coli* and Gram positive *B. subtilis* were also used as controls to infect 3-D cells; however neither bacterium had detectable intracellular growth by 24 hpi, further suggesting that a bacterial specific active invasion mechanism is necessary for bacterial internalization into 3-D cells. These growth curves also demonstrate the ability of the *Salmonella* mutants to invade and survive within 3-D cells for 24 hours, as they are not killed by the gentamicin in the extracellular media as observed with *E. coli* and *B. subtilis* (Figure 2D and Figure 5B). From these data, we conclude that SPI-1, SPI-2, and the flagellar secretion system are not necessary for invasion into 3-D HT-29 cells, but are required for wildtype levels of invasion and intracellular replication.

## Discussion

In previous work, we established and characterized a 3-D organotypic model of human large intestinal epithelium from the colonic epithelial cell line HT-29 utilizing the cellular suspension RWV bioreactor system [32], [36]. HT-29 cells grown in three-dimensions in the RWV displayed numerous characteristics relevant to their *in vivo* tissue counterparts, demonstrating the potential of utilizing RWV-derived 3-D cells as surrogate models to elucidate cellular mechanisms and responses to conditions encountered in the human gastrointestinal tract, such as during an enteric pathogen infection [32], [36]. We previously reported that an *invA Salmonella* mutant (which is defective for invasion of monolayers) invaded into 3-D HT-29 cells to similar levels as wildtype, and to our knowledge, this was the first report to suggest that the SPI-T3SS may not be the main determinant for invasion of *Salmonella* into *in vitro* models of human intestinal epithelium [32], [43]. While intriguing, our previous findings did not definitively preclude a role for the SPI-1 T3SS in intestinal invasion, nor did we apply the 3-D large intestinal model to investigate the role of the two other T3SSs in *Salmonella*, the SPI-2 encoded T3SS and the *flhDC* regulated flagellum secretory system. In this report, we fundamentally expanded our studies to show that the 3-D model of large intestinal epithelium displays multicellular complexity relevant to the normal parental tissue *in vivo*, including formation of M-cells/M-like cells, enterocytes and Paneth cells. We subsequently applied these highly differentiated 3-D intestinal cultures as human surrogate infection models to elucidate the role of all characterized *Salmonella* T3SSs in the early stages of human enteric salmonellosis. Specifically, we infected 3-D HT-29 and monolayer cells with various *Salmonella* mutants carrying single (SPI-1 or SPI-2), double (SPI-1/2) and a complete T3SS knockout (SPI-1/SPI-2: *flhDC*), respectively. We observed substantial differences in the roles of the T3SSs during invasion into and growth within the 3-D HT-29 cells as compared to monolayers, but notably, we identified similar key phenotypic responses between the 3-D cells following *Salmonella* challenge with those reported during the *in vivo* infection process.

The initial key difference that we observed in response to infection between 3-D large intestinal cells and established data utilizing monolayers, was the significant increase in invasion of a SPI-1 encoded T3SS mutant into the 3-D cells (Figure 2A and B), a mutant previously characterized to be invasion defective in monolayers [43], [44]. The SPI-1 mutant lacking the entire SPI-1 coding region displayed lower levels of invasion compared to the *invA* T3SS mutant in both culture systems, and this could be a result of the absence of the entire SPI-1 PAI (Pathogenicity Island) in the SPI-1 mutant, which could potentially contain effectors aiding in bacterial invasion. Surprisingly, a SPI-1/2 double mutant and a SPI-1/2: *flhDC* triple mutant, a strain lacking all characterized T3SS secretion systems, also invaded into the 3-D intestinal cells at similar levels (Figure 2E). While the TTSS mutants were able to invade 3-D cells, the invasion levels were significantly lower compared to wildtype. These data suggest that TTSSs contribute to a more productive bacterial invasion of 3-D cells, but an unidentified bacterial factor(s) is contributing to majority of *Salmonella* invasion into 3-D cells. We are currently attempting to identify the bacterial and/or host factors that are the major regulators of invasion for *Salmonella* into human 3-D intestinal cells. However, once intracellular, each single, double and triple T3SS mutant was unable to grow to levels similar to that of wildtype (Figure 5B), suggesting that each T3SS system is necessary for successful replication of *Salmonella* inside 3-D intestinal cells. Interestingly, even though the SPI-1 mutant still contains the SPI-2 system and its encoded effectors predicted to be responsible for intracellular survival, the SPI-1 mutant did not replicate as well as wildtype once inside the host cells. These data are consistent with work utilizing monolayers showing that a *Salmonella invA* mutant was unable to grow after induced invasion of epithelial cells [68]. We are currently characterizing the role of SPI-2 and its secreted effectors in *Salmonella* intracellular trafficking and replication within our 3-D intestinal models.

Until recently, the paradigm for *Salmonella* entry into human intestinal epithelial cells was based upon results from monolayer cell cultures and non-human animal models [6], [10], [17], [18], [19], [20], [21], [22], [26], [27]; however our work with highly differentiated 3-D human intestinal epithelial cells cultivated in the RWV bioreactor demonstrated infection phenotypes more closely related to those observed *in vivo* [28], [31], [32], [36], [49]. The RWV bioreactor system is only one example of how advancements in technology facilitate the improvement of tissue engineered model systems to more accurately mimic *in vivo* conditions in the native host, thus allowing researchers to study host-pathogen interactions in an accessible, reproducible, and tractable *in vitro* system [22], [23], [24], [26], [36], [39]. However, as previously discussed, choice of host species has been shown to greatly influence the outcome of a *Salmonella* infection, and several studies utilizing a variety of advanced model systems have shown that T3SSs are necessary for *Salmonella* invasion and infection of these models [17], [18], [19], [21], [22], [23], [26], [39]. Recently however, non-human models studying *Salmonella* pathogenesis are in agreement with our findings reported in this study, that SPI-1 is not critical for the invasion of host cells. One such example was conducted by Coombes *et al.*, 2005, wherein this group utilized a novel explant/re-implantation calf ileal loop model to investigate the role of SPI-1 and SPI-2 during the later stages of a *Salmonella* infection [69]. Interestingly, the SPI-1 *Salmonella* mutant not only infected the tissues of the small intestine, but by 5 days post infection, severe inflammation, submucosal edema, necrosis, vascular thrombosis, and villus atrophy had also occurred. In a separate study, Desin *et al.*, 2009, observed colonization of a *Salmonella enterica* subsup. *Enterica* serovar Enteritidis strain lacking SPI-1 in the cecum of chickens [70]. In a similar example, the fowl-adapted *Salmonella* strain, *Salmonella enterica* serovar Gallinarum, was also able to invade animal cells independently of SPI-1 [71]. Moreover, a *Salmonella* Typhimurium SPI-1 mutant was recovered at comparable levels to the wildtype strain from the intestine, intestinal contents, Peyer's patches, lymph nodes, and spleen of mice following oral infection [49]. Recently, using an *in vitro* polarized co-cultured human intestinal cell culture model, wildtype *Salmonella* Typhimurium and mutants lacking multiple SPI-1 effectors showed similar translocation of host cells [39]. Results from these studies and the naturally occurring *Salmonella* infection, demonstrate the lack of dependence of *Salmonella* on SPI-1 to cause disease in human and animal hosts. These studies not only corroborate our findings regarding the lack of *Salmonella* dependence on SPI-1 to invade our 3-D organotypic intestinal epithelial models, but also demonstrate the urgent need for development of more *in vivo-*like cell culture systems and their use in combination with animal models to better understand the underlying mechanisms of disease.

In an attempt to identify the bacterial mechanism(s) playing a role in invasion of our 3-D intestinal epithelial cells in the absence of SPI-1, we constructed a *Salmonella* mutant absent for all characterized T3SSs that could be utilized to secrete effectors aiding in invasion (SPI-1 T3SS, SPI-2 T3SS, and the flagellar pore). Interestingly, invasion of the SPI-1/2/flagellar secretory mutant (defective for all T3SSs) still occurred in our 3-D intestinal model (Figure 2), alluding to the potential of an uncharacterized or undiscovered mechanism(s) utilized by *Salmonella* for invasion into 3-D intestinal cells and possibly during infection as well. Currently, 12 different *Salmonella* pathogenicity islands (PAIs) have been identified based on genetic classifications and characteristics of PAIs, however many of the identified PAI-encoded genes only have predicted putative functions with no clear role in *Salmonella* pathogenesis [72]. Interestingly, several PAIs are predicted to encode functions that have the capacity to assist in *Salmonella* invasion of 3-D intestinal cells, with numerous genes encoding for pili and fimbrae [73], [74]. Similar with published reports, we have already demonstrated that type 1 pili encoded by the *fim* operon is not necessary for invasion into 3-D intestinal cells (Figure 3C and D), however in work done by others, SPI-7 was shown to contain a putative type IVB pilus that could be aiding in bacterial invasion in this study [57], [72]. Moreover, SPI-6 and SPI-10 encode for fimbrae, and both were shown to be critical in bacterial invasion and intracellular survival [72], [75], [76], [77], [78]. However, the genomes of *Salmonella* serovars contain large numbers of fimbrial operons located outside of PAIs, any of which could potentially be responsible for the SPI-1 independent invasion of 3-D intestinal cells, in turn making the identification of the exact invasion mechanism(s) quite challenging [79], [80]. Moreover, in recent work, SPI-4 was demonstrated to encode a functional T1SS used to secrete effectors necessary for bacterial adhesion to epithelial cells [81]. To further complicate identification of the mechanism(s) responsible for *Salmonella* invasion into our 3-D intestinal cells, the epithelial cells themselves will also likely express and display surface structures necessary for bacterial invasion. Accordingly, both host and bacterial factor(s) have to be investigated, and we are currently examining several of these mechanisms in the attempt to identify the means of *Salmonella* invasion into our 3-D intestinal epithelial cells.

In this study, we utilized a rotating cell culture system that allows mammalian cells to be grown under physiological levels of fluid shear stress that help facilitate the formation of 3-D cellular aggregates possessing structural and functional characteristics similar to the *in vivo* parental tissues [36]. As previously observed *in vivo* where *Salmonella* was able to infect a host in the absence of a functional SPI-1 T3SS, we have shown analogous results in our 3-D organotypic models of human intestinal epithelium. We also show that *Salmonella* invasion into our 3-D intestinal models occurred in the absence of all characterized *S. typhimurium* type three secretory systems (SPI-1 T3SS, SPI-2 T3SS, and flagellar pore) that could be transporting bacterial effectors assisting in invasion into host epithelial cells. Moreover, our results indicate that invasion of 3-D intestinal cells by wildtype and SPI mutants was a bacterial active process, whereas non-invasive bacterial strains did not invade the 3-D cells, even those cells with known absorptive properties, including M-cells/M-like cells and enterocytes. Studies are on-going in our lab to define the molecular mechanism(s) of *Salmonella* SPI-1 independent epithelial cell invasion into our 3-D intestinal models.

The work presented here reveals many key similarities between the response of in vitro 3-D models of human intestinal epithelium and their parental tissues *in vivo* during a *Salmonella* infection, highlighting the potential of these organotypic models to 1) serve as powerful tools to facilitate the dissection of the molecular mechanisms of human enteric salmonellosis, 2) provide an *in vivo* like system to study other gastrointestinal pathogens, and 3) serve as models for the development of novel strategies to combat infectious disease.

## Materials And Methods

### Mammalian Cell Lines and Bacterial Strains

Bacterial strains used in this study are described in Table S1. All bacterial strains were grown in Lennox broth (L broth) as described below [82]. The human colonic epithelial cell line, HT-29, was obtained from the American Type Culture Collection (ATCC) [32], [83], [84]. The cells were cultured in a specialized growth medium comprised of a triple-sugar minimal essential medium α–L-15 base supplemented with 10% fetal bovine serum, designated as GTSF-2 [85], and incubated at 37°C at 5% CO_2_ environment.

### Antibodies and Reagents

Antibodies against specified antigens were obtained from the following sources: Sialyl Lewis A (MC-551; Kamiya), polyclonal *Salmonella* O antisera (BD Difco), monoclonal *Salmonella* antibody (MAI-83451; Affinity Bioreagents), syndecan-1 (MCA681T; AbD seroTec), Phospholipase A2 (PLA2) (ab58375; Abcam), ITF goblet cell marker (15C6; Santa Cruz), and Alexa Fluor 633 phalloidin (A22284; Invitrogen). Fluoresbrite plain YG 2.00 micron microspheres (fluorescent beads) were obtained from Polysciences, Inc. (18338). For mammalian cell viability, 0.4% trypan blue (Sigma) was used at a 1∶1 ratio with cells suspended in cell culture media.

### Growth of 3-D intestinal cells in the RWV

The development of 3-D intestinal tissue models was performed as previously described [31], [32]. In brief, HT-29 epithelial cells were initially grown as monolayers in tissue culture flasks, trypsinized, and resuspended in fresh medium at a density of 2×10^6^ cells/ml. Cells were then introduced into RWV bioreactors (Synthecon, Inc.) together with collagen-coated Cytodex-3 microcarrier beads (Pharmacia), resulting in a final ratio of ∼10 cells/bead [85]. Cells were cultured in the RWV bioreactors in GTSF-2 at 37°C and 5% CO_2_ with rotation at 20 rpm. Fresh medium was replenished after 5 days and then every 24 hours thereafter until harvest of the cultures at 18-23 days.

### Bacterial invasion and intracellular growth assays

HT-29 monolayers were grown in GTSF-2 medium in a 24-well plate at a concentration of ∼1.2 million cells/plate. For 3-D aggregates, cells were harvested from the bioreactor and seeded into 24 well plates at a concentration of ∼19 million cells/plate. For infections, all bacterial strains were grown shaking overnight in L broth medium at 37°C. Bacteria were back-diluted 1∶200 and grown with aeration until the bacterial growth reached OD 600 of ∼0.8. Bacteria were washed twice with Dulbecco's PBS (D-PBS) and used to infect mammalian cells at a multiplicity of infection (m.o.i.) of 10 for 1 h, whereupon infected cells were washed three times with D-PBS and incubated in medium containing 100 µg/ml gentamicin for 3 hour. The cells were then washed three times with D-PBS and fresh medium containing 7.5 µg/ml gentamicin was added. At indicated times post-infection (p.i.), media was removed, cells were washed with PBS, lysed with 500 µl of 0.1% Triton-X 100, and a fraction of the lysate was plated on L agar to enumerate colony-forming units (CFU). For heat killed *Salmonella* studies, bacteria were taken from the back diluted *Salmonella* cultures, washed 3 times with D-PBS, resuspended in D-PBS, heated at 90°C for 30 minutes, and used to infect mammalian cells as with live *Salmonella*.

### Confocal Microscopy

The 3-D aggregates were harvested from the bioreactor and seeded in a 24 well plate at a concentration of approximately 19 million cells/plate. Infections were done either using same protocol as invasion/intracellular growth assays described above, or an established protocol described by Mortimer *et al.*, which allows for a shorter and synchronized infection [56]. In brief, *Salmonella* overnight cultures grown in LB shaking at 37°C were back diluted 1∶200 in LB and grown shaking until they reached an OD of ∼1.2. Bacteria were washed 2 times in D-PBS and used to infect mammalian cells at an m.o.i. of 10 for 5 minutes, whereupon host cells washed 3 times and media was replaced with media containing 100 µg/ml gentamicin. At indicated times p.i., 3-D aggregates were fixed in 4.0% paraformaldehyde in D-PBS for 30 minutes at room temperature. For staining, cells were washed three times with 0.1% Triton X-100 in PBS and blocked for 1 hour with blocking buffer (D-PBS, 0.1% Triton X-100, 1% BSA). Blocking buffer with primary antibody (concentration antibody dependent) was added to the cells for 1 hour at 37°C. Cells were then rinsed three times with D-PBS and incubated for 1 hour with secondary antibody diluted 1∶1000 in blocking buffer. Aggregates were rinsed with D-PBS and mounted with Pro-Long Gold Anti-fade with DAPI (Invitrogen). Samples were analyzed with the Carl Zeiss 510 Inverted confocal microscope using a 100× objective and ZEN Light Edition 2009 software. Quantitation of *Salmonella* colocalization with the various host markers was determined by fixing cells at indicated times p.i., staining the cells with the designated host and *Salmonella* specific antibodies, then counting the number of bacteria that localized with the markers per 35-50 bacteria in at least three independent experiments. For differential immunofluorescence staining of extracellular and intracellular bacteria, infections were carried out as described above in the bacterial invasion and intracellular growth assays section and staining was done as described in results section and in published reports [46].

### Statistical analysis

Data sets in triplicate were analyzed using Microsoft Excel software to calculate the Student's unpaired t-test for independent samples. P-values of <0.05 (*) and <0.001 (**) were considered significant and highly significant, respectively.

## Supporting Information

Text S1 **Experimental Method.**
(DOC)

Figure S1 **Cellular localization of** ***Salmonella*** **and T3SS mutants during infection of 3-D HT-29 cells.** Confocal immunofluorescence microscopy images (100×) of 3-D HT-29 aggregates with wildtype, SPI-1, and SPI-1/2 *Salmonella* mutants for 1 h, and fixed 3 hpi. Cells were stained with a monoclonal anti-*Salmonella* antibody (blue; extracellular bacteria), washed, permeabilized, and stained with a polyclonal anti-*Salmonella* serum (blue and red; extracellular and intracellular bacteria) and phalloidin to stain host cell actin (green). Arrow heads point to bacteria that stain only red (intracellular bacteria).
(TIF)

Figure S2 **Localization of 2 µm beads upon exposure to 3-D intestinal cells.**
Confocal immunofluorescence microscopy z-stack frames (100×) from 3-D HT-29 aggregates exposed with 2 µm fluorescent beads (red) at a concentration of 10 beads/cell for 1 h, fixed at 3 h post exposure, and counter-stained with phalloidin to visualize host cell actin (green).
(TIF)

Figure S3 **Intracellular growth and localization of** ***Salmonella*** **during infection of 3-D intestinal cells.**
A) Intracellular growth profiles of wildtype *Salmonella* and SPI-1 mutant at 3 hpi (solid bars) and 24 hpi (outlined bars) comparing 3-D cells infected with *Salmonella* grown to an OD of 0.8 and infected at an m.o.i of 10 for 1 hour (black bars) and 3-D cells infected with *Salmonella* grown to an OD of 1.2 and infected at an m.o.i. of 10 for 5 minutes (grey bars). Data represents the average of at least three independent experiments from separate batches of cells (N = 3). B) Confocal immunofluorescence microscopy images (100×) of 3-D HT-29 aggregates infected with wildtype *Salmonella*, fixed at 15 mpi, and stained with the host early endosomal marker anti-EEA1 antibody (61046; BD Transduction Laboratories) (red), anti-*Salmonella* antibody (green), and DAPI (blue). C) Confocal immunofluorescence microscopy images (100×) of 3-D HT-29 aggregates infected with wildtype *Salmonella*, fixed at 2 hpi, and stained with the host lysosomal marker anti-LAMP-1 antibody (H4A3; DSHB) (red), anti-*Salmonella* antibody (green), and DAPI (blue). D) RT-PCR of wildtype (lanes 1-4) and SPI-1 mutant (lanes 5-8) *Salmonella invA* expression levels at a bacterial growth OD600 of 0.6 (lanes 1 and 5), 0.8 (lanes 2 and 6), 1.2 (lanes 3 and 7), and 1.8 (lanes 4 and 8). Lane 9 is a negative control of a PCR reaction containing no cDNA. Expression of *16s rRNA* was used as loading control.
(TIF)

Figure S4 **M-cell marker sialyl Lewis A antigen is expressed on the surface of 3-D HT-29 aggregates.**
Confocal immunofluorescence microscopy z-stack frames (100×) from 3-D HT-29 aggregates infected with wildtype *Salmonella* at 5 mpi, fixed, and stained with anti-sialyl Lewis A antibody (red), anti-*Salmonella* antibody (green), and DAPI (blue).
(TIF)

Figure S5 **Localization of** ***Salmonella*** **SPI-1 mutant to the M-cell marker sialyl Lewis A antigen in 3-D HT-29 aggregates.**
Confocal immunofluorescence microscopy z-stack frames (100×) from 3-D HT-29 aggregates infected with SPI-1 *Salmonella* mutant at 5 mpi, fixed, and stained with anti-sialyl Lewis A antibody (red), anti-*Salmonella* antibody (green), and DAPI (blue).
(TIF)

Figure S6 **Differential expression patterns of enterocyte and M-cell markers in 3-D HT-29 cells.**
Confocal immunofluorescence microscopy images (100×) of 3-D HT-29 aggregates fixed and stained with an enterocyte marker antibody, anti-syndecan-1 (green), and M-cell maker antibody, anti-sialyl Lewis A (red).
(TIF)

Table S1 **Bacterial strains used in this study.**
(TIF)
