# Supplementary material for: Analysis of Interactions of Salmonella Type Three Secretion Mutants with 3-D Intestinal Epithelial Cells
Source: PLoS One. 2010 Dec 29;5(12):e15750. doi: 10.1371/journal.pone.0015750 (PMC3012082; doi:10.1371/journal.pone.0015750)
Supplement: Text S1 — Experimental Method. (DOC) [file pone.0015750.s001.doc]

**SUPPORTING TEXT 1**

**Experimental Method**

**Reverse transcription polymerase chain reaction (RT-PCR) analysis**

Wildtype and the SPI-1 *Salmonella* mutant were back-diluted 1:200 from overnight shaking cultures and grown to the indicated OD600. A sample was removed from each indicated OD to obtain a concentration of 1.2x10^9^ bacteria/ml, and sample was pelleted, resuspended in RNAII later, and stored at -80°C. After collection of all samples, samples were thawed, pelleted, resuspended in 2mg/ml TE lysoszyme, and incubated at room temperature for 15 minutes. Total RNA was prepared from by using RNeasy Miniprep Kit (Qiagen) and RNase-free DNase (1unit/mg RNA) according to manufacturer’s protocol. CDNA was synthesized from RNA samples using MonsterScript 1^st^-Strand Kit (EPICENTRE biotechnologies) according to manufacturer’s protocol. RT-PCR was performed using *Taq* 2X Master Mix (New England BioLabs), with the following PCR conditions: 25 cycles of denaturation at 94°C for 30 sec, annealing at 55°C for 30 sec, and extension at 72°C for 30 sec. An additional extension was performed at 72°C for 5min. Primers used include: *invA*-F (ACCAAAGGACACGACTTCATCGGA), *invA*-R (GACGCAGCTGTTGAACAACCCATT), *16S rRNA*-F (GTAACGGCTCACCAAGGCGACGATCCCTAG), and *16S rRNA*-R (CTTCGCCACCGGTATTCCTCCAGATCTCTAC). The same primers and cDNA were used to run a quantitative RT-PCR using IQ Syber Green Supermix (Biorad) on the Eppendorf RealPlex Mastercycle.

**SUPPORTING REFERENCES**

1. Gulig PA, Curtiss R, 3rd (1987) Plasmid-associated virulence of *Salmonella typhimurium*. Infect Immun 55:2891-2901.

2. Galan JE, Curtiss R 3rd (1989) Cloning and molecular characterization of genes whose products allow Salmonella typhimurium to penetrate tissue culture cells.

Proc Natl Acad Sci U S A 86: 6383-7.

3.Jones BD, Falkow S (1994) Identification and characterization of a *Salmonella*

*typhimurium* oxygen-regulated gene required for bacterial internalization. Infect Immun 62:3745-3752.

4. Suvarnapunya AE, Stein MA (2005) DNA base excision repair potentiates the protective effect of *Salmonella* Pathogenicity Island 2 within macrophages. Microbiology 151:557-567.

5. Coombes BK, Coburn BA, Potter AA, Gomis S, Mirakhur K, Li Y, and Finlay BB (2005) Analysis of the contribution of *Salmonella* pathogenicity islands 1 and 2 to enteric disease progression using a novel bovine ileal loop model and a murine model of infectious enterocolitis. Infect Immun 73:7161-7169.

6. Yanagihara S, Iyoda S, Ohnishi K, Iino T, and Kutsukake K (1999) Structure and transcriptional control of the flagellar master operon of *Salmonella typhimurium*. Genes Genet Syst 74:105-111.
