# Supplementary figures and images for: Analysis of Interactions of Salmonella Type Three Secretion Mutants with 3-D Intestinal Epithelial Cells
Source: PLoS One. 2010 Dec 29;5(12):e15750. doi: 10.1371/journal.pone.0015750 (PMC3012082; doi:10.1371/journal.pone.0015750)

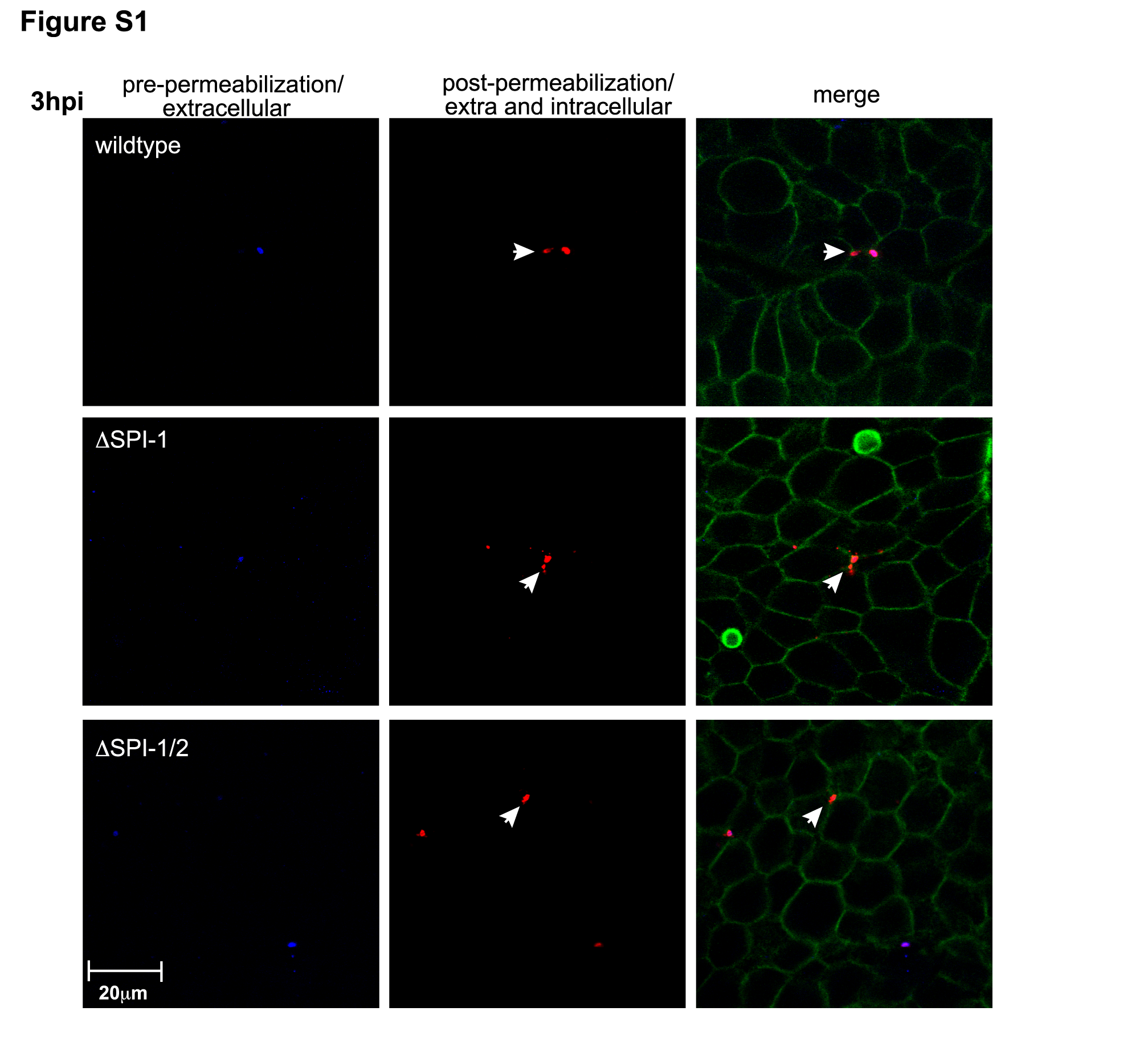

Supplement: Figure S1 — Cellular localization of Salmonella and T3SS mutants during infection of 3-D HT-29 cells. Confocal immunofluorescence microscopy images (100×) of 3-D HT-29 aggregates with wildtype, SPI-1, and SPI-1/2 Salmonella mutants for 1 h, and fixed 3 hpi. Cells were stained with a monoclonal anti-Salmonella antibody (blue; extracellular bacteria), washed, permeabilized, and stained with a polyclonal anti-Salmonella serum (blue and red; extracellular and intracellular bacteria) and phalloidin to stain host cell actin (green). Arrow heads point to bacteria that stain only red (intracellular bacteria). (TIF) [file pone.0015750.s002.tif]

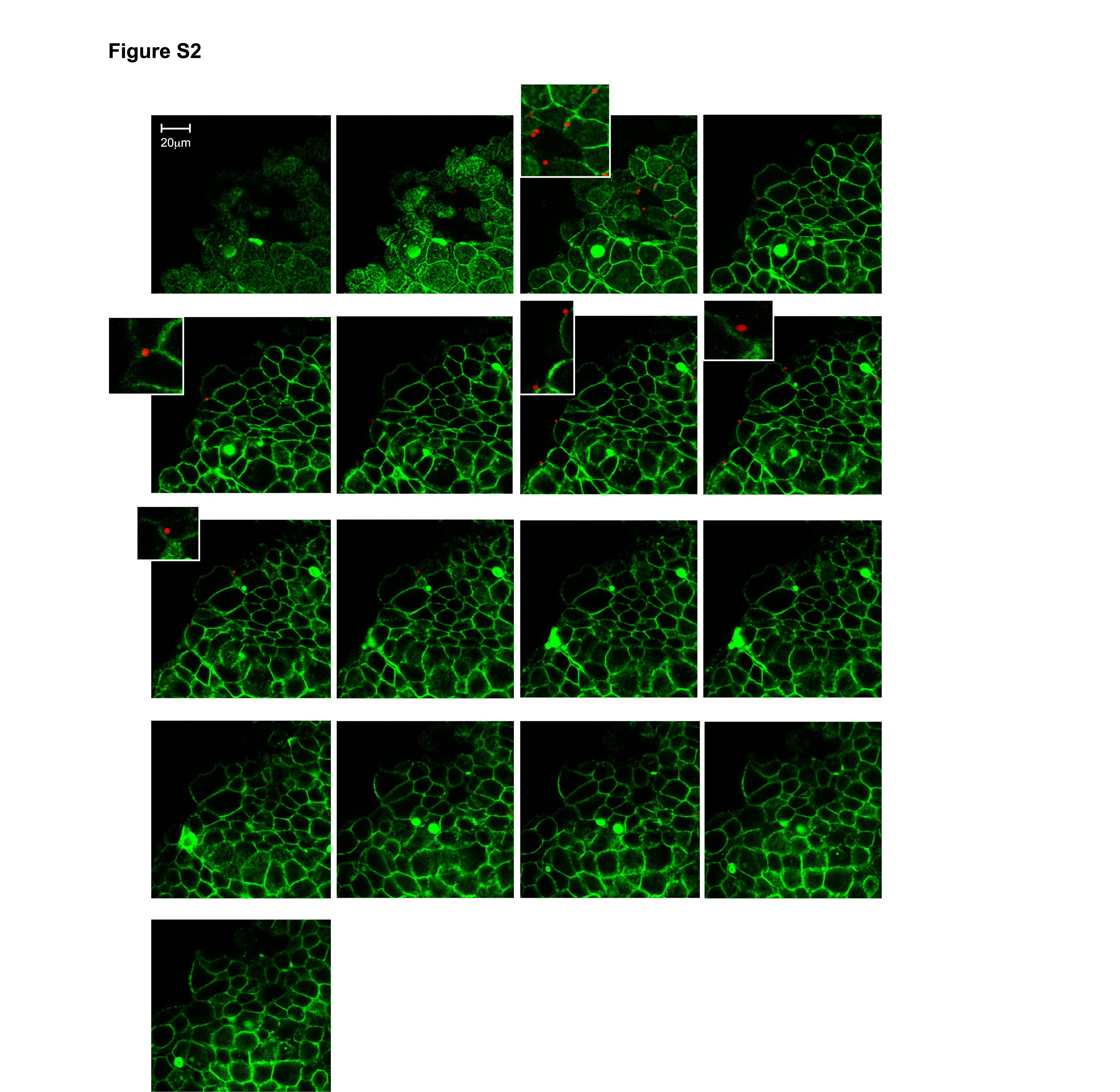

Supplement: Figure S2 — Localization of 2 µm beads upon exposure to 3-D intestinal cells. Confocal immunofluorescence microscopy z-stack frames (100×) from 3-D HT-29 aggregates exposed with 2 µm fluorescent beads (red) at a concentration of 10 beads/cell for 1 h, fixed at 3 h post exposure, and counter-stained with phalloidin to visualize host cell actin (green). (TIF) [file pone.0015750.s003.tif]

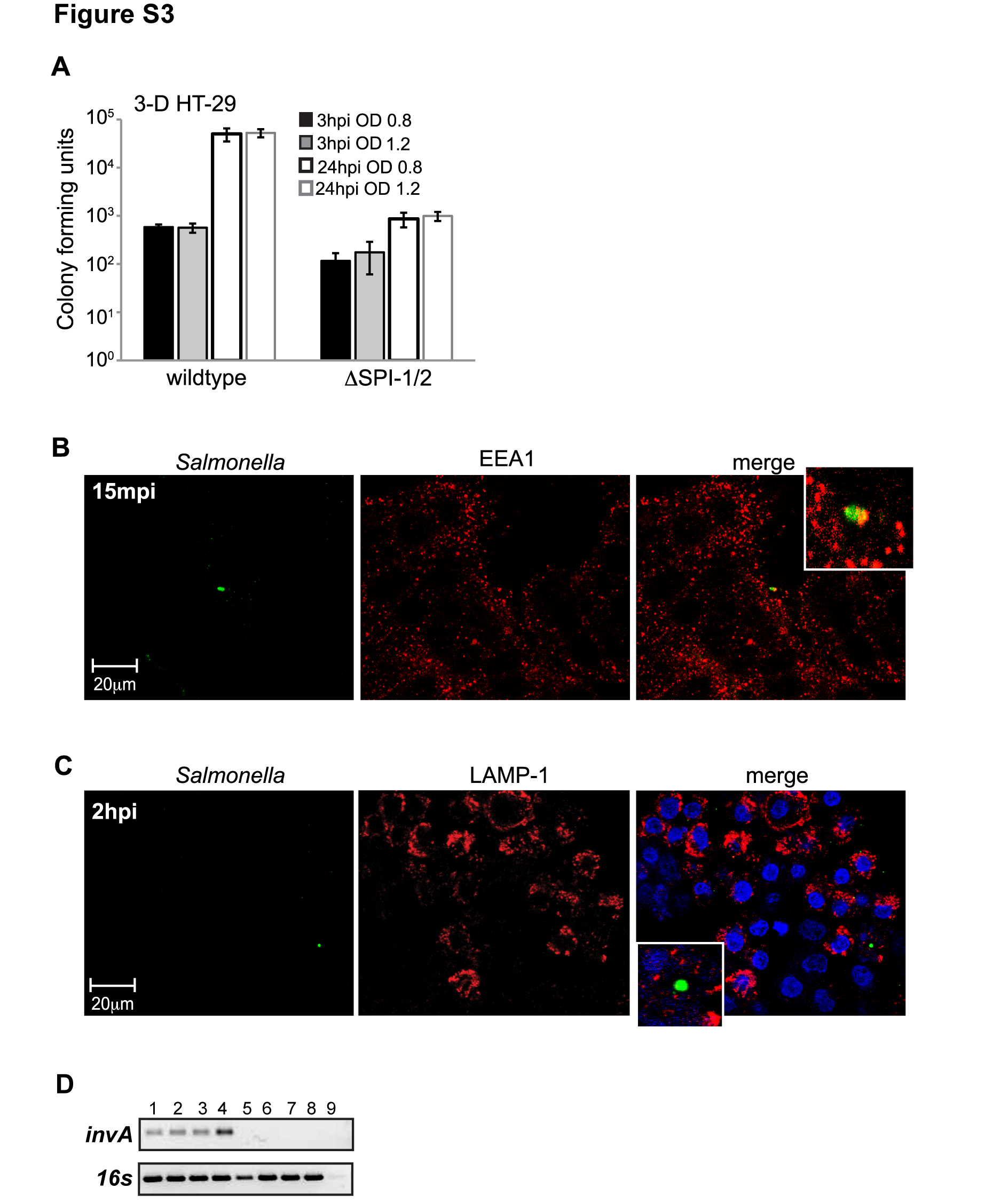

Supplement: Figure S3 — Intracellular growth and localization of Salmonella during infection of 3-D intestinal cells. A) Intracellular growth profiles of wildtype Salmonella and SPI-1 mutant at 3 hpi (solid bars) and 24 hpi (outlined bars) comparing 3-D cells infected with Salmonella grown to an OD of 0.8 and infected at an m.o.i of 10 for 1 hour (black bars) and 3-D cells infected with Salmonella grown to an OD of 1.2 and infected at an m.o.i. of 10 for 5 minutes (grey bars). Data represents the average of at least three independent experiments from separate batches of cells (N = 3). B) Confocal immunofluorescence microscopy images (100×) of 3-D HT-29 aggregates infected with wildtype Salmonella, fixed at 15 mpi, and stained with the host early endosomal marker anti-EEA1 antibody (61046; BD Transduction Laboratories) (red), anti-Salmonella antibody (green), and DAPI (blue). C) Confocal immunofluorescence microscopy images (100×) of 3-D HT-29 aggregates infected with wildtype Salmonella, fixed at 2 hpi, and stained with the host lysosomal marker anti-LAMP-1 antibody (H4A3; DSHB) (red), anti-Salmonella antibody (green), and DAPI (blue). D) RT-PCR of wildtype (lanes 1-4) and SPI-1 mutant (lanes 5-8) Salmonella invA expression levels at a bacterial growth OD600 of 0.6 (lanes 1 and 5), 0.8 (lanes 2 and 6), 1.2 (lanes 3 and 7), and 1.8 (lanes 4 and 8). Lane 9 is a negative control of a PCR reaction containing no cDNA. Expression of 16s rRNA was used as loading control. (TIF) [file pone.0015750.s004.tif]

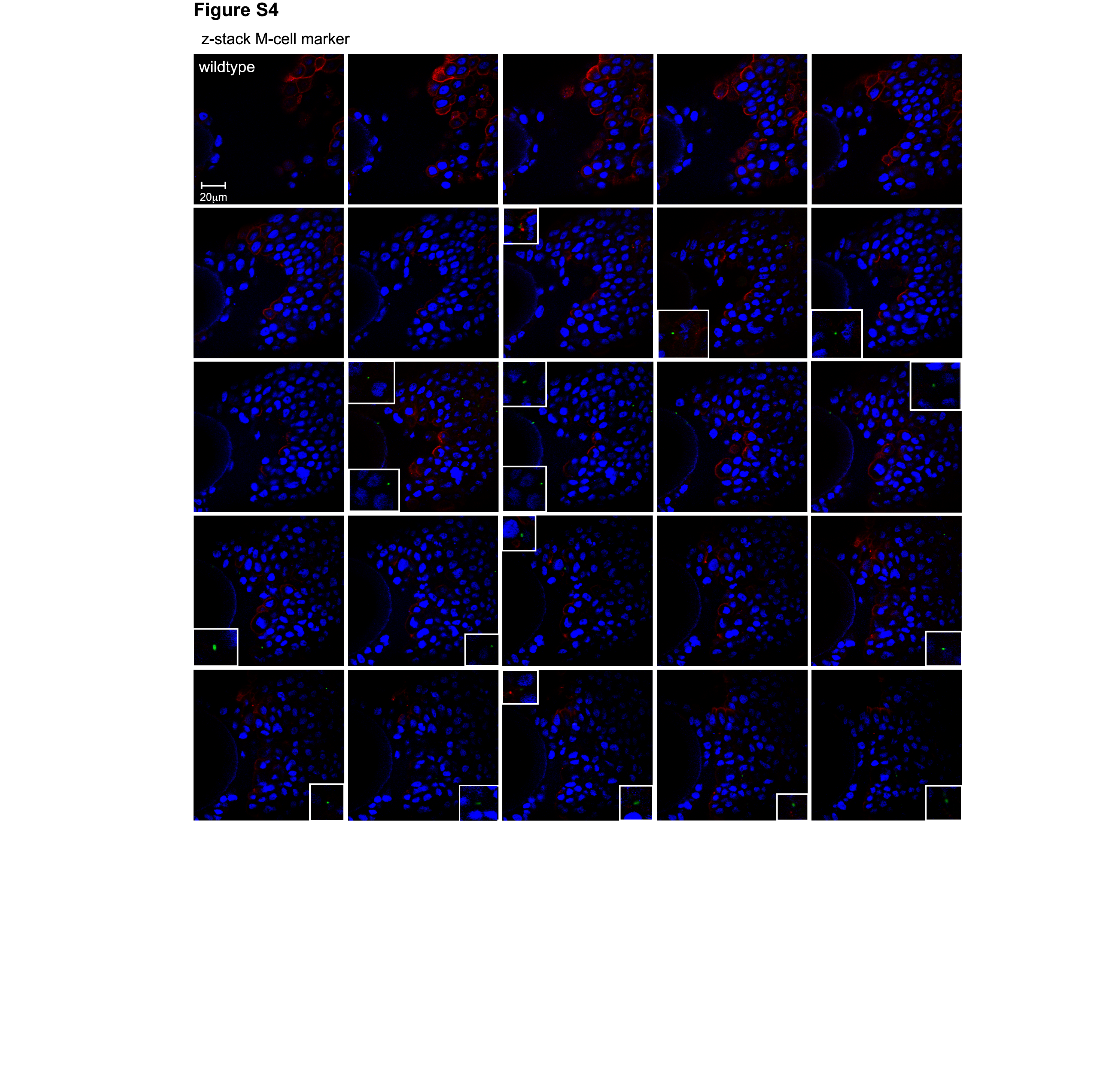

Supplement: Figure S4 — M-cell marker sialyl Lewis A antigen is expressed on the surface of 3-D HT-29 aggregates. Confocal immunofluorescence microscopy z-stack frames (100×) from 3-D HT-29 aggregates infected with wildtype Salmonella at 5 mpi, fixed, and stained with anti-sialyl Lewis A antibody (red), anti-Salmonella antibody (green), and DAPI (blue). (TIF) [file pone.0015750.s005.tif]

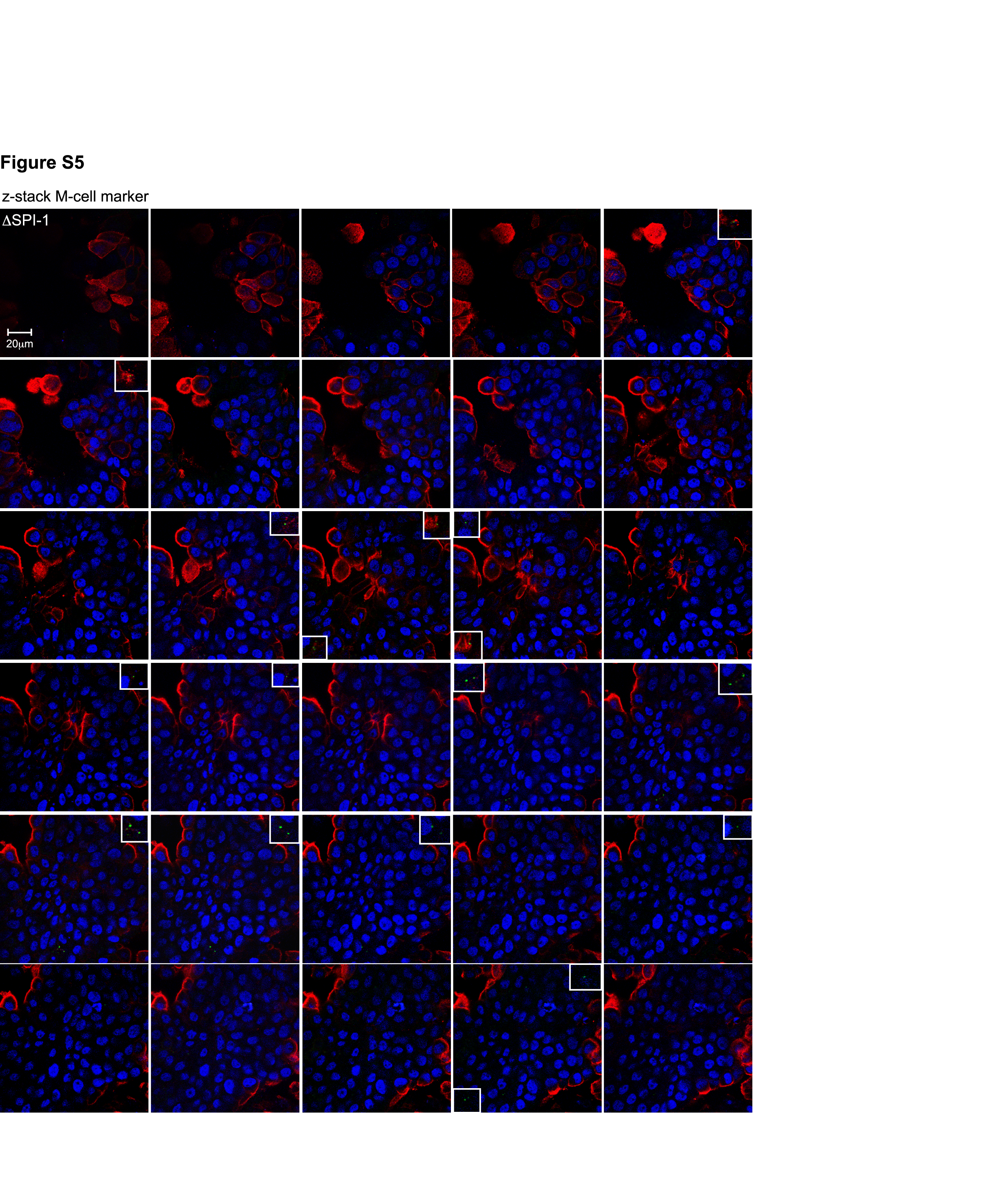

Supplement: Figure S5 — Localization of Salmonella SPI-1 mutant to the M-cell marker sialyl Lewis A antigen in 3-D HT-29 aggregates. Confocal immunofluorescence microscopy z-stack frames (100×) from 3-D HT-29 aggregates infected with SPI-1 Salmonella mutant at 5 mpi, fixed, and stained with anti-sialyl Lewis A antibody (red), anti-Salmonella antibody (green), and DAPI (blue). (TIF) [file pone.0015750.s006.tif]

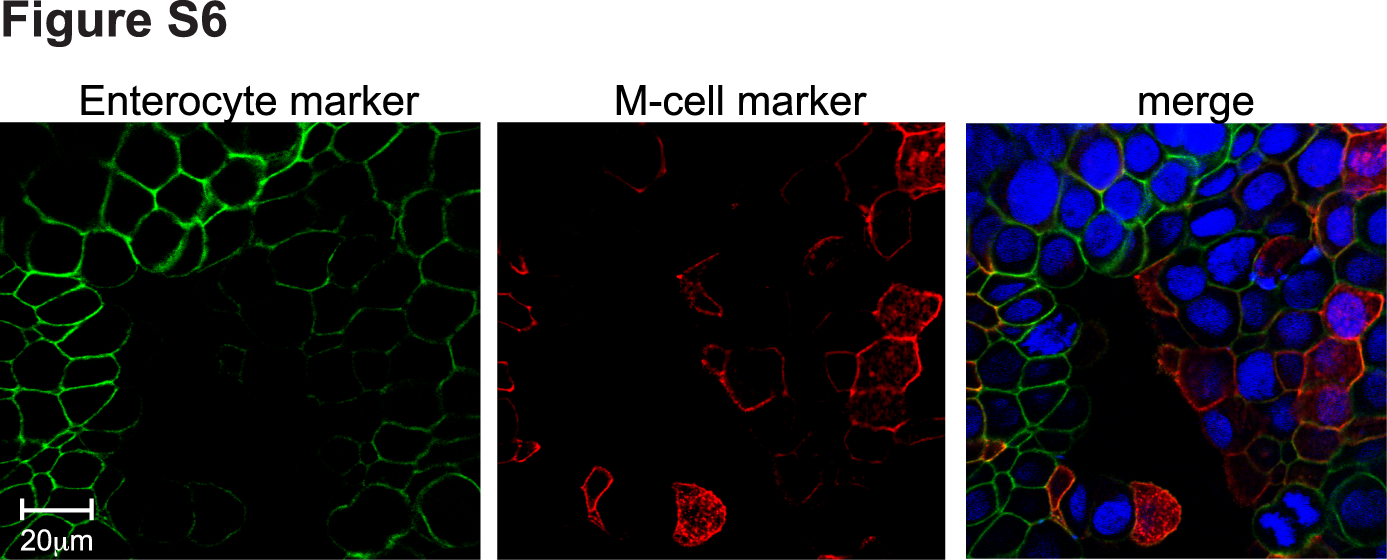

Supplement: Figure S6 — Differential expression patterns of enterocyte and M-cell markers in 3-D HT-29 cells. Confocal immunofluorescence microscopy images (100×) of 3-D HT-29 aggregates fixed and stained with an enterocyte marker antibody, anti-syndecan-1 (green), and M-cell maker antibody, anti-sialyl Lewis A (red). (TIF) [file pone.0015750.s007.tif]

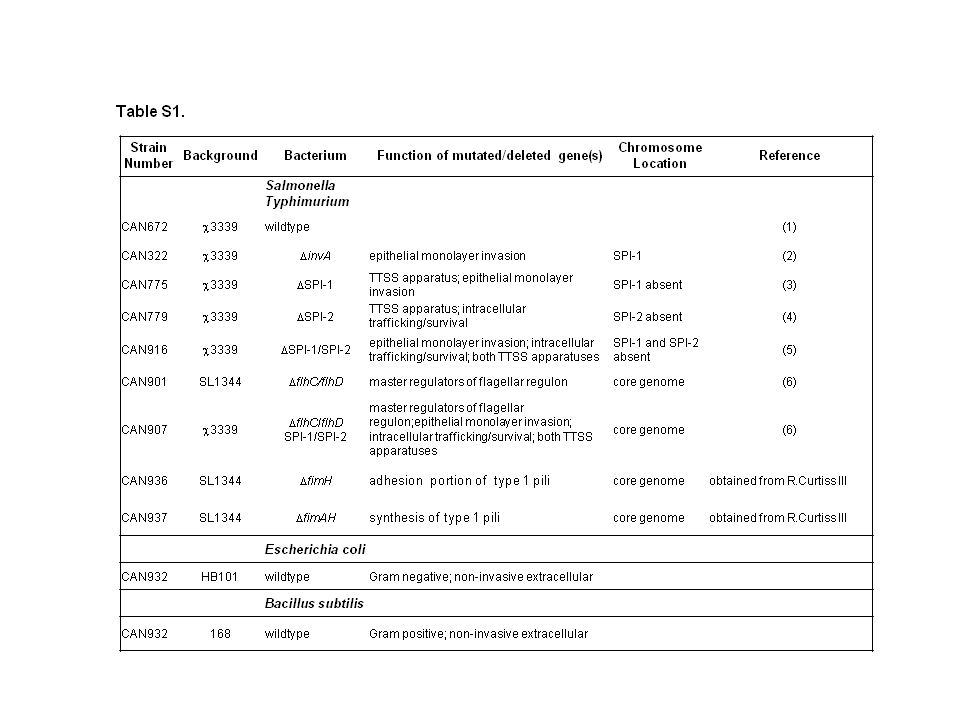

Supplement: Table S1 — Bacterial strains used in this study. (TIF) [file pone.0015750.s008.tif]
